# Schulschließungen als ethische Herausforderung

**DOI:** 10.1007/s35834-022-00364-4

**Published:** 2022-10-10

**Authors:** Dagmar Schulze Heuling, Christoph Helm

**Affiliations:** 1 Staatswissenschaftliche Fakultät, Universität Erfurt, Nordhäuser Str. 63, 99089 Erfurt, Deutschland; 2 Linz School of Education, Johannes Kepler Universität Linz, Altenberger Straße 69, 4040 Linz, Österreich

**Keywords:** COVID-19, Ethik, Schulschließungen, Deontologie, Utilitarismus, COVID-19, Ethics, School Closures, Deontology, Utilitarianism

## Abstract

Die COVID-19-Pandemie hat weltweit viele Staaten zu drastischen Maßnahmen greifen lassen. Unabhängig von der epidemiologischen Wirkung ist unumstritten, dass diese Maßnahmen erhebliche negative Auswirkungen u. a. auf die Schulleistungen, die psychosoziale und die physische Gesundheit der Kinder und Jugendlichen hatten. Angesichts ihrer Folgen evozieren solche Eingriffe die Frage nach ihrer ethischen Rechtfertigbarkeit. Der vorliegende Beitrag führt in die gängigsten ethischen Theorien ein und diskutiert darauf aufbauend die ethische Bewertung der COVID-19-bedingten Schulschließungen.

**Zusatzmaterial online:**

Zusätzliche Informationen sind in der Online-Version dieses Artikels (10.1007/s35834-022-00364-4) enthalten.

## Abstract (en)

The COVID-19 pandemic has led many countries around the world to take drastic measures. Regardless of the epidemiological impact, it is undisputed that these measures have had significant negative effects on scholastic attainment, psychosocial and physical health of children and adolescents. Given their consequences, such interventions evoke the question of their ethical justifiability. This article introduces the most common ethical theories and, building on them, discusses the ethical evaluation of the COVID-19-related school closures.

## Einleitung

Die COVID-19-Pandemie hat weltweit viele Staaten zu drastischen Maßnahmen greifen lassen. Zuvor ungekannte und unvorstellbare Einschränkungen griffen tief in alle Lebensbereiche ein. Großveranstaltungen und Gottesdienste wurden verboten, Restaurants und Kultureinrichtungen mussten schließen, Bewohner*innen von Pflegeeinrichtungen wurden weitgehend isoliert. Private physische Kontakte wurden drastisch eingeschränkt, und vielerorts galten Ausgangssperren. Schulen bildeten keine Ausnahme. Sie wurden zeitweilig für den Präsenzbetrieb geschlossen.

Unabhängig von der epidemiologischen Wirkung ist unumstritten, dass mit diesen Maßnahmen auch erhebliche negative Auswirkungen einhergingen. Angesichts ihrer Folgen evozieren solche Eingriffe die Frage nach ihrer ethischen Rechtfertigbarkeit. Was ist noch erlaubt, um die Gesundheit zu schützen, und welche Maßnahmen sind tabu, selbst wenn man damit ein Menschenleben retten könnte? Im Fall von Schulschließungen muss diese Prüfung besonders kritisch ausfallen, weil Kinder und Jugendliche durch das SARS-CoV-2-Virus selbst kaum gefährdet sind (Ludvigsson 2020; Hoang et al. 2020; Filippatos et al. 2021). Die ihnen auferlegten Lasten schützen daher primär nicht sie selbst, sondern – hoffentlich – andere.

Dieser Artikel betrachtet mit der Aussetzung des Präsenzunterrichts, wie sie in Österreich und Deutschland praktiziert wurde, einen kleinen Ausschnitt der Maßnahmen zur Pandemieeindämmung und ihrer komplexen Auswirkungen. Es liegt auf der Hand, dass damit kein Anspruch auf Vollständigkeit verbunden ist. Vielmehr ist unser Anliegen, einen Beitrag zur Diskussion der ethischen Bewertung der Folgen der Aussetzung des Präsenzunterrichts zu leisten.

Dazu gehen wir wie folgt vor: Zunächst stellen wir nationale und internationale empirische Befunde zu den Auswirkungen von Schulschließungen zusammen. Dabei konzentrieren wir uns auf die Aspekte Schülerleistungen (Abschn. 2.1), Psychosoziale Folgen (Abschn. 2.2) und die physische Gesundheit (Abschn. 2.3). Ebenso betrachten wir die Effekte auf das Infektionsgeschehen (Abschn. 2.4). Das anschließende Kapitel (Abschn. 3) führt in die gängigsten ethischen Theorien ein und bildet damit die Grundlage für die ethische Bewertung der Schulschließungen, die in Abschn. 4 erfolgt. Der Beitrag schließt mit einer zusammenfassenden ethischen Bewertung von Schulschließungen.

## Auswirkungen von Schulschließungen

### Schülerleistungen

Erste Meta-Reviews und -analysen von Helm et al. (2021), Betthäuser et al. (2022), Hammerstein et al. (2021), König und Frey (2022), Patrinos und Donnelly (2021) und Zierer (2021) geben einen Überblick über Leistungsstudien, die die Frage nach Lerneinbußen und neuer Bildungsungleichheit aufgrund der pandemiebedingten Schulschließungen im Frühjahr 2020 zu beantworten versuchen.^1^ Nimmt man die Befunde der in den Überblicksarbeiten enthaltenen Studien (z. B. Schult et al. 2022; Engzell et al. 2021; Rose et al. 2021)^2^ zusammen, so kann festgehalten werden, dass die bestehende Datenlage die vielfach erwarteten Lerneinbußen mehrheitlich bestätigt. Auch die befürchtete Bildungsbenachteiligung von Schüler*innen aus sozioökonomisch schlechter gestellten Familien wird von vielen Studien aufgezeigt. Dabei zeigt sich die Tendenz, dass in höheren Schulstufen und in der Domäne Lesen eine Zunahme der sozio-ökonomischen Benachteiligung aufgrund von Schulschließungen weniger wahrscheinlich ist als im Primarstufenbereich und in der Domäne Mathematik.

Allerdings muss an dieser Stelle auch auf die substanzielle Zahl von Studien, die keine relevanten Lerneinbußen und keine signifikante, zusätzliche Bildungsbenachteiligung durch Schulschließungen im Frühjahr 2020 beobachten können, verwiesen werden (DACH-Raum (Deutschland, Österreich, Schweiz), z. B.: Depping et al. 2021; Förster et al. 2021; international, z. B.; Gore et al. 2021; van der Velde et al. 2021)^3^. Da sich alle vorliegenden Studien auf das Frühjahr 2020 beziehen, ist es jedoch unwahrscheinlich, dass darin das wahre Ausmaß der Lerneinbußen und Bildungsbenachteiligung umfassend abgedeckt wird. Die negativen Effekte späterer Schulschließungen ebenso wie die langfristigen Folgen gilt es zu erforschen.

Schließlich liegt empirisches Wissen hauptsächlich für die Domänen Mathematik, Erstsprache und Lesen vor, sodass über Lerneinbußen in anderen Schulfächern keine wissenschaftlich abgesicherten Aussagen gemacht werden können. Gerade aber für Schulfächer wie Musik und Sport sind negative Effekte sehr wahrscheinlich, da in diesen Fächern der Unterricht vergleichsweise lange ausfiel. Ebenfalls fehlen Studien, die überfachliche Fähigkeiten wie soziales Lernen und kommunikative Fähigkeiten in den Blick nehmen. So wurde in Österreich die mündliche Matura, und damit eine zentrale Prüfungs- aber auch Lerngelegenheit, während der Schuljahre 2019/20 sowie 2020/21 ausgesetzt.

Negative Effekte der Schulschließungen lassen sich nicht nur an den Outcomes der Schüler*innen ablesen, sondern auch an der Qualität der Lernumgebung, konkret der Schul- und Unterrichtsqualität, die den Schüler*innen geboten wurde. Aus Sicht von Lehrkräften und Schulleitungen sind Merkmale der Beschaffenheit von Schule, insbesondere auch Unterrichtsqualitätsmerkmale wie die Schülerzentrierung durch die Schulschließungen signifikant zurückgegangen (Huber et al. im Druck). Im Gegensatz zu Schulen in privilegierter Lage berichten insbesondere Schulen mit höherer sozialer Belastung, das heißt einem höheren Anteil von Schüler*innen aus sozioökonomisch und kulturell benachteiligten Familien, von einem stärkeren Rückgang in der Schul- und Unterrichtsqualität (ebd.; siehe auch Bremm und Racherbäumer 2020). Anzunehmen ist auch, dass sich der während der Pandemie zu beobachtende, steigende Personalausfall an Schulen mit den bekannten Folgen etwa für Betreuungsschlüssel, zusätzliche Angebote und fachfremden Unterricht insbesondere an sozial benachteiligten Schulen negativ auf den Lernfortschritt auswirkt (Huber et al. im Druck).

### Psychosoziale Folgen

Überblicksarbeiten zu Effekten der Schulschließungen existieren nicht nur für den Bereich des fachlichen Kompetenzerwerbs der Schüler*innen, sondern auch für Aspekte der psychosozialen Gesundheit. So haben Schlack et al. (2020) in ihrer sehr frühen Überblicksarbeit (eingereicht 20.08.2020) bestehende Studien zu den Auswirkungen der Schulschließungen im Frühjahr 2020 auf die Gesundheit der Kinder und Jugendlichen zusammengetragen. Sie fassen die Befundlage wie folgt zusammen:

Bei Kindern und Jugendlichen traten Symptome von Angst und Depression sowie eine geminderte Lebensqualität auf. Die Schließungen der Betreuungs- und Bildungseinrichtungen und der damit einhergehende Verlust der gewohnten Tagesstruktur, Kontaktabbrüche und dem eigenständigen Lernen zu Hause stellten erhebliche Herausforderungen für betroffene Kinder und deren Familien dar. Räumliche Enge und fehlende Ausweichmöglichkeiten während der Eindämmungsmaßnahmen konnten außerdem zu erhöhtem familiärem Stress und gehäufter familiärer Aggression sowie zu häuslicher Gewalt führen. (S. 23)

Wie für die Meta-Studien zu den Lerneinbußen, ist auch für die Überblicksarbeit von Schlack et al. (2020) anzunehmen, dass die gesammelten Befunde die negativen Effekte der Schulschließungen letztlich unterschätzen, da mittlerweile in mehreren Studien belegt ist, dass der psychische Stress bzw. die Belastung der psychischen Gesundheit mit Fortlauf der Pandemie unter den Schüler*innen wie den Eltern deutlich anstieg (Kaman et al. 2021).

### Folgen für die physische Gesundheit

Viele Studien und erste Meta-Analysen verweisen nicht nur auf emotionale und verhaltensbezogene Probleme (z. B. Angstzustände, depressive Symptome), sondern auch auf negative Auswirkungen auf das körperliche Wohlergehen von Schüler*innen (Viner et al. 2021; Tan 2021; OECD 2021). Die Befunde betreffen eine große Bandbreite negativer Konsequenzen für die physische Gesundheit. So ist mittlerweile gut belegt, dass die mit den Kontaktbeschränkungen und dem Wegfall des Sportunterrichts verbundene Verringerung der körperlichen Aktivität bei Jugendlichen zu Übergewicht geführt hat (z. B. Viner et al. 2021; Tan 2021). Generell gilt Bewegungsmangel als ein führender Risikofaktor für nicht ansteckende Erkrankungen (Guthold et al. 2018), von Herz-Kreislauf-Erkrankungen über Diabetes bis hin zu Krebs (Warburton et al. 2010; Sallis et al. 2016).

Nachgerade kontraproduktiv wirkt sich die mit dem Präsenzverbot, aber auch mit den Abstands- und Maskengeboten verbundene pauschale Reduktion der Pathogenexposition aus. Insbesondere für die kindliche Immunentwicklung ist eine maßvolle Exposition gegenüber Krankheitserregern unverzichtbar (Apostol et al. 2020; Georgountzou und Papadopoulos 2017). Radikale Expositionsprophylaxe begünstigt nicht nur Immunpathologien, durch den Nachholeffekt bei anderen Infektionen kam es auch zu einer Überlastung pädiatrischer Stationen (Ärzteblatt 2021).

Besonders nachdenklich stimmen Studien, die die Effekte von Schulschließungen auf die Lebenserwartung der jungen Generation untersuchen. Da die Anzahl besuchter Schuljahre – insbesondere in der Grundschule – mit Gesundheit und hoher Lebenserwartung korreliert, schätzen Christakis et al. (2020) mittels komplexer Modellberechnungen, dass weltweit 0,8 (Europa) bis zu 13,8 (USA) Millionen Lebensjahre durch die Schulschließungen verloren gingen. Für den DACH-Raum liegen ebenfalls Studien vor, die einen deutlichen Zusammenhang zwischen (einem höheren) Bildungsabschluss und (einer geringeren) Sterblichkeitsrate belegen (z. B. Rau et al. 2008; Grigoriev und Doblhammer 2019; Klein 1999), sodass die von Christakis et al. (2020) geschätzten Effekte von Schulschließungen auch für den DACH-Raum von Bedeutung sein dürften.

Vor dem Hintergrund der dargestellten Befunde resümieren Gesundheitsforscher*innen (Viner et al. 2021; Liu et al. 2020), dass diese Ergebnisse in künftige politische Entscheidungen einbezogen werden müssen. Sie sollten die Basis für die Abwägung von Risiken der Übertragung des Virus durch Kinder und Jugendliche im Schulalter gegen die (gesundheitlichen) Nachteile von Schulschließungen bilden.

### Effekte von Schulschließungen auf die Anzahl von SARS-CoV-2-Infektionen

Die Motivation und Rechtfertigung für Schulschließungen liegt in ihrem unterstellten, dämpfenden Einfluss auf Infektionen mit SARS-CoV‑2. Daher schließt sich hier die Frage an, ob Schulschließungen überhaupt ein wirksames Mittel sind, um die Infektionszahlen einzudämmen. Review-Studien (z. B. Walsh et al. 2021; Tan 2021) zeigen, dass die Befundlage zu dieser Frage heterogen und inkonsistent ist. Auf Ebene von Einzelstudien beobachten Staguhn et al. (2021) positive Effekte von Schulschließungen auf COVID-19-Infektionsraten. Dagegen liegen zwei Studien (von Bismarck-Osten et al. 2021; Isphording et al. 2021) aus Deutschland vor, die keinen Anstieg der Infektionsraten bei Schulwiedereröffnungen beobachten konnten. Letztere werden von internationalen Review-Studien (Ludvigsson 2020; Irfan et al. 2021) gestützt, die zu dem Schluss kommen, dass es unwahrscheinlich ist, dass Kinder die Treiber der Pandemie sind. Die Studienautor*innen argumentieren daher, dass (a) die Schließung/Wiedereröffnung von Schulen und Kindergärten keinen relevanten Einfluss auf die COVID-19-Sterblichkeitsrate bei älteren Menschen haben dürfte und (b) für Kinder (< 10 Jahre) es weitgehend sicher ist, die Schule zu besuchen. Überhaupt geht von COVID-19 keine nennenswerte Gesundheitsgefahr für Kinder aus, wie Meta-Studien belegen (z. B. Hoang et al. 2020; Liu et al. 2020). Dagegen sind Schulschließungen, wie in Abschn. 2.2 und 2.3 dargestellt, nicht ohne gesundheitliche Folgen (Tan 2021; Viner et al. 2020). Für die ethische Beurteilung von Schulschließungen hat dieses uneindeutige Bild bzgl. der Effekte von Schulschließungen auf die COVID-19-Infektionsraten jedoch eine eindeutige Implikation: Die Tatsache, dass viele Studien keinen oder allenfalls einen geringen infektionseindämmenden Effekt von Schulschließungen konstatieren, bedeutet, dass ihr Nutzen unklar ist. Ein (noch) nicht empirisch belegbarer Nutzen muss in jeder rationalen Auseinandersetzung als hypothetisch bewertet werden.

Zugleich kommen die genannten Studien zu dem Schluss, dass Kinder und Jugendliche nicht die primären Überträger*innen der Krankheit sind; insbesondere nicht junge Kinder. So kommen bspw. Irfan et al. (2021) in ihrer Meta-Review zum Schluss, dass Kinder unter 10 Jahren eine deutlich geringere Anfälligkeit für COVID-19 haben als Erwachsene. Weil Covid-19 für Kinder und Jugendliche jedenfalls nicht gefährlicher ist als andere Atemwegsinfektionen (Ludvigsson 2020; Hoang et al. 2020), werben viele Ärztinnen und Ärzte daher auch öffentlich für den Besuch von Schulen und Kitas.^4^

Vor dem Hintergrund der umfassenden Befundlage zu negativen Auswirkungen der Schulschließungen auf die kognitive, psychische und physische Entwicklung von Kindern und Jugendlichen evozieren solche Eingriffe die Frage nach ihrer ethischen Rechtfertigbarkeit. Was ist noch erlaubt, um die Gesundheit von Risikogruppen zu schützen, wenn dies auf Kosten anderer Gruppen passiert? Zur Erörterung dieser schwierigen Frage, führen wir im nächsten Kapitel in die gängigsten ethischen Theorien ein, um so die Basis für die Bewertung der Schulschließungen, die in Abschn. 4 erfolgt, zu legen.

## Ethik

Die philosophische Ethik^5^ wird traditionell in drei große Strömungen unterteilt: Deontologie, Konsequentialismus und Tugendethik. Der zentrale Unterschied zwischen diesen Denkschulen besteht darin, was sie als relevanten Anknüpfungspunkt eines moralischen Urteils betrachten: die fragliche Handlung, das Ergebnis der Handlung oder die handelnde Person. Auf letztere stellt die Tugendethik ab. Ihr zufolge soll eine Handlung aus tugendhaften Charaktereigenschaften (die daher eingeübt werden sollen) heraus vorgenommen werden. Weil deren Vorhandensein aus der Ferne nicht seriös beurteilt werden kann, bleiben tugendethische Überlegungen im Weiteren außen vor.

### Deontologie

Deontologische Ethiken gehen davon aus, dass es eine Pflicht gibt, moralische Normen zu beachten. Ihr Urteil beruht auf der (Nicht‑)Übereinstimmung der zu beurteilenden Handlung mit der relevanten Norm (vgl. Alexander und Moore 2021). In der Praxis führt das zu einem weitreichenden Gleichklang ethischer Urteile. Andere Menschen zu bestehlen, zu verletzen oder sogar zu töten ist unethisch. Doch da über die Quellen, den Umfang und die Natur der ethischen Normen Uneinigkeit besteht, kommen deontologische Ethiken in Konfliktfällen mitunter zu unterschiedlichen Ergebnissen.

Kant betrachtete die Vernunft als Ausgangspunkt aller Ethik. Der berühmte kategorische Imperativ soll jener vernünftige Wille des autonomen Subjekts sein, der alle Handlungen leitet – und zwar explizit „ohne auf die daraus erwartete Wirkung Rücksicht zu nehmen“ (Kant 1785, S. 402). Es ist nur folgerichtig, dass in der kantischen Ethik die so gewonnenen Normen absolut gelten – wie in jüngerer Zeit wieder bei Rawls (1972). Für die Intuition, um eines sehr großen Vorteils willen eine kaum merkliche Regelverletzung in Kauf zu nehmen, ist hier kein Raum, allein schon deshalb, weil sich die Qualität und Größe von Vorteil und Regelverletzung nicht aus der Vernunft bestimmen lassen.

Dieser Gedanke ist logisch bestechend. Kant hat ihn allerdings sehr weit getrieben. So verurteilt er eine Lüge sogar dann, wenn man damit den Aufenthaltsort eines Freundes vor Häschern verbirgt (Kant 1797). Dem Einwand, dass das Verrat und gerade unethisch sei, versucht der Ansatz von Ross Rechnung zu tragen. Ross greift eine insbesondere von Prichard (1912) verfolgte Idee auf und bestimmt die ethischen Überzeugungen reflektierter Menschen als Ausgangspunkt seiner Theorie. Aus diesen gewinnt er einen kleinen Katalog sogenannter prima-facie Pflichten, darunter die Pflicht zum Wohltun (beneficience) und zum Nicht-Schädigen (non-maleficience, Ross 1930).

Diese Pflichten stehen in keiner festen Hierarchie zueinander. Allerdings ist zu vermuten, dass die Pflicht des Nicht-Schädigens auch für Ross schwerer wiegt als andere (vgl. Skelton 2022). Auch für andere deontologische Ansätze haben solche negativen oder absoluten Pflichten Priorität. Für sie ist es falsch, einen Menschen zu töten, selbst wenn man z. B. durch dessen Organe anderen das Leben retten kann (Thomson 2008). Dissens besteht allerdings in der Frage, ob und wie positive Pflichten (etwa anderen Menschen zu helfen) Berücksichtigung erfahren. Für Kant waren sie mangels vernünftiger Bestimmbarkeit unvollkommen und können einen Verstoß gegen die Vernunft nicht rechtfertigen. Folgt man hingegen Ross’ Ansatz, ist bei einem eklatanten Missverhältnis der negativen Konsequenzen – etwa im Fall der erwähnten Notlüge oder wenn man Bauholz vom Nachbargrundstück entwendet, um damit ein ertrinkendes Kind zu retten – die Verletzung negativer Gebote, wie nicht zu lügen oder zu stehlen, nicht nur erlaubt, sondern nachgerade eine ethische Forderung.

### Konsequentialismus

Konsequentialistischen Theorien knüpfen ihr Urteil an die Folgen einer Handlung. Zu dieser Gruppe zählen ethische Egoismen in der Tradition des Epikur oder der Stoa, die das (langfristige) Selbstinteresse des Individuums in den Mittelpunkt stellen. Bekannter sind aber wohl utilitaristische Theorien, die darauf abzielen, die durch eine Handlung bewirkten positiven Folgen zu maximieren.

Die klassische Form des Utilitarismus wurde von Bentham formuliert. Für ihn werden Menschen durch zwei Meister regiert: durch *pain* und *pleasure* (Bentham 1843, S. 118f). Darin stützt er sich vermutlich auf Beccaria, der auch die Sentenz vom größten Glück der größten Zahl prägte. Ethisch ist für Bentham diejenige Handlung, die für die Gesamtheit der betrachteten Menschen das Positive befördert. Dies nennt er „principle of utility“ (ebd.).

Die Details utilitaristischer Ethik waren von Anfang an umstritten. Wie das Positive definiert ist (Glück, Nutzen, Wohlfahrt, erfüllte Präferenzen, etc.), wie die Aggregation erfolgen soll, ob Anknüpfungspunkt die konkrete oder die abstrakte Handlung ist (Akt- vs. Regelutilitarismus), wie streng das Maximierungsgebot auszulegen ist, wird nach wie vor unterschiedlich beurteilt. Sehr grob lässt sich jedoch festhalten, dass das Hervorbringen positiver Folgen zentrales Urteilskriterium utilitaristischer Ethiken ist (vgl. Driver 2014; Hübner 2014).

Praktisch gesprochen: Man darf das Holz zur Rettung des Kindes entwenden und den Freund durch eine Lüge schützen. Fraglich ist allerdings, wo die Grenzen des Utilitarismus liegen. Wenn man nur genügend Menschen retten kann, ist bei stringenter Anwendung des Utilitarismus sogar das Töten eines Menschen nicht nur nicht verboten, sondern nachgerade eine Forderung der Moral. Das wiederum ist ein Anknüpfungspunkt für Kritik: Der Utilitarismus kann das Individuum nicht vor einer radikalen Entrechtung, schlimmstenfalls gar Opferung für andere schützen. Zugleich kann die Verpflichtung zur Reduktion von Elend bzw. zur Mehrung von Wohl einen Menschen schnell überfordern. Ähnlich wie bei der Deontologie dienen unterschiedliche Hilfsannahmen dazu, solche Ergebnisse auszuschließen.

## Ethische Bewertung

Ein ethisches Urteil kommt zustande, indem man eine ethische Theorie auf eine lebensweltliche Situation anwendet. Somit hängt das Urteil sowohl von der gewählten Theorie ab als auch von der Einschätzung der lebensweltlichen Situation; beides kann Quelle von Differenzen sein. Das gilt auch für die Diskussion der ethischen Implikationen von Schulschließungen.

### Vorannahmen

Um die Komplexität der Diskussion etwas zu reduzieren, setzen wir einige – weitestgehend unkontroverse – Annahmen voraus. So gehen wir davon aus, dass COVID-19 eine schwerwiegende Erkrankung ist. Die durchschnittliche Infection-Fatality-Rate wurde in einem Preprint des WHO-Bulletin im Oktober 2020 mit 0,27 % beziffert (Ioannidis 2020). Ebenso sehen wir es als gesichert an, dass das Risiko, schwer an COVID-19 zu erkranken oder gar zu versterben, stark mit dem Lebensalter und Vorerkrankungen zusammenhängt. Daraus folgt, dass eine SARS-CoV-2-Infektion für gesunde Kinder keine nennenswerte Gesundheitsgefahr darstellt (vgl. auch Ludvigsson 2020; Hoang et al. 2020; Filippatos et al. 2021).

### Deontologische Überlegungen

Aus einer deontologischen Perspektive ist die Analyse anhand der Frage aufzuschlüsseln, ob die Schulschließungen auf Grund einer moralischen Pflicht bzw. eines moralischen Rechts der Kinder und Jugendlichen moralisch geboten, zulässig oder verboten waren. Geboten hätten sie etwa sein können, wenn sie zum Schutz der Gesundheit von Kindern und Jugendlichen selbst erforderlich waren. Da allerdings von einer SARS-CoV-2-Infektion für gesunde Kinder keine nennenswerte Gesundheitsgefahr ausgeht, kann dieses Argument Schulschließungen nicht rechtfertigen. Sie waren zum Schutz der Gesundheit von Kindern und Jugendlichen nicht geboten und wegen ihres Verletzungscharakters folglich unzulässig.

Allerdings sind Kinder und Jugendliche nicht die einzigen Menschen, die potenziell von einer COVID-19-Erkrankung betroffen sein können. Zweifelsohne ist der Schutz Dritter, die von Kindern und Jugendlichen angesteckt werden könnten und im Falle einer Erkrankung ungleich gefährdeter wären als diese, nicht nur ein menschliches Bedürfnis. Es handelt sich auch um eine positive ethische Pflicht. Insofern ist zu prüfen, ob der Schutz dieser Menschen die Schulschließungen trotz der mit ihnen einhergehenden Schäden rechtfertigen kann.

Grundsätzlich ist hier eine hohe argumentative Hürde zu überwinden. Die Schulschließungen haben Kinder und Jugendliche körperlich und seelisch verletzt, die Lernrückstände wirken sich, wie oben umfassend beschrieben, ebenfalls in vielerlei Hinsicht negativ aus. Es hat folglich eine aktive Verletzung einer negativen Pflicht stattgefunden. Dem gegenüber steht die Erfüllung einer positiven Pflicht, der Schutz von Menschen vor einer schweren Krankheit. Schlicht ausgedrückt stellt sich die Frage, wie sehr man der einen Gruppe schaden darf, um der anderen Gruppe zu helfen.

Eine sehr konsequente deontologische Theorie lehnt diesen Gedanken rundweg ab und betrachtet Schulschließungen, die dem Schutz der Gesundheit Dritter dienen, als ethisch unzulässig. Egal, wie löblich oder menschlich verständlich das Ziel ist, darf man nach dieser Ansicht nicht einige Menschen für andere opfern und ihre Gesundheit, ihr Wohlergehen oder ihre Zukunftschancen aufs Spiel setzen.

Schließt man sich hingegen einer vermittelnden Position an, sind zwei Aspekte zu beachten: Erstens das Verhältnis der beiden Pflichten zueinander und zweitens die Wirksamkeit der fraglichen Maßnahme. Die Abwägung des Schadensverbots und Rettungsgebots kann zu unterschiedlichen Ergebnissen führen. Angesichts bedrückender Bilder von Menschen, die um ihr Leben ringen, liegt es nahe, das Retten von Menschenleben in diesem Fall als die stärkere Pflicht zu betrachten. Das muss allerdings gleichermaßen für Kinder und Jugendliche gelten. Die Schäden, die durch die Schulschließungen angerichtet worden sind, wirken sich auch verkürzend auf ihre Lebenszeit aus. Nur dann, wenn man die verlorenen Lebensjahre der Kinder als kategorisch verschieden von jenen der Erwachsenen betrachtet, kann überhaupt ein Weg zur deontologischen Rechtfertigung von Schulschließungen eröffnet sein.

Hinsichtlich des zweiten Aspekts, der Wirksamkeit von Schulschließungen, überwiegt hingegen die Skepsis, ob das Schließen der Schulen tatsächlich einen nennenswerten Beitrag zur Senkung von Infektionszahlen leisten kann. Unabhängig davon, ob sich diese Einschätzung bewahrheitet oder als falsch herausstellt, führen Zweifel an der Wirksamkeit von Schulschließungen dazu, dass sie ethisch zunehmend fragwürdig werden. Je ungewisser der gesundheitsschützende Effekt für die Bevölkerung, desto schwieriger ist es, die voraussehbaren Schäden zu rechtfertigen.

Zusammenfassend ist somit festzuhalten, dass Schulschließungen aus einer deontologischen Perspektive nur schwerlich gerechtfertigt werden können.

### Utilitaristische Überlegungen

Die Pandemie hat ein unvorstellbares Ausmaß an Leid mit sich gebracht. Es übersteigt menschliche Fähigkeiten, dies gänzlich zu verhindern. Nach utilitaristischer Auffassung lautet der ethische Auftrag jedoch, so zu handeln, dass das unter den gegebenen Umständen bestmögliche Ergebnis erzielt wird. Je nach Theorie kann das die Erfüllung von Präferenzen sein, die Minimierung von Leid oder Maximierung von Glück.

Betrachtet man erfüllte individuelle Präferenzen als das moralisch relevante Ziel, so sind einheitliche Lösungen – gleichviel, ob sie im Schließen von Schulen oder in der Verpflichtung zum Schulbesuch bestehen – schon aus logischen Gründen vielfach ethisch suboptimal, weil damit auf die Realisierung eines höheren Maßes an Präferenzerfüllung verzichtet wird. Selbst wenn die getroffene Entscheidung die Präferenz der Mehrheit widerspiegelt, ist die Minderheit, deren Präferenz nicht erfüllt wurde, häufig groß. Lösungen, die den Individuen einen größeren Entscheidungsspielraum belassen, führen in der Regel zu einem höheren Grad an Präferenzerfüllung. Auch in der Frage der Schulschließungen wäre es aus präferenzutilitaristischer Sicht vorzugswürdig, den Schulbesuch freizustellen und die Entscheidung darüber den Betroffenen zu überlassen.

Ganz anders stellt sich die Situation dar, wenn das Ziel die Minimierung von Leid ist. Dazu müssen zunächst die unterschiedlichsten Formen von Leid quantifiziert und gegeneinander aufgerechnet werden. In einigen Fällen ist das einfach, so etwa im obigen Beispiel der Requirierung von Rettungsgerät. Dass das Fehlen des zur Rettung benutzten Holzes zu Konsequenzen führt, die schwerer wiegen als der Tod eines Menschen, kann wohl nur in philosophischen Abhandlungen passieren. In der Realität ist ein derartiger Verlauf nahezu ausgeschlossen, sodass das Retten des Menschen fraglos den höheren Nutzen stiftet bzw. mehr Leid verhindert.

Doch je komplexer die Situation, je unterschiedlicher die Belastungen und Vorteile, je ungewisser der Wirkungszusammenhang, desto schwieriger wird es, die einzelnen Faktoren gegeneinander aufzurechnen. Wie ist psychisches Leid gegenüber physischem Leid zu gewichten? Was ist schlimmer: ein Auge zu verlieren oder eine Hand? Hinzu kommt, dass die erforderliche Abwägung bisweilen als abstoßend empfunden wird. Doch eine nutzen- oder glücksorientierte Ethik muss angeben, ob zwei verlorene Lebensjahre schwerer wiegen, wenn der Verlust einen hochaltrigen, womöglich mehrfach vorerkrankten Menschen trifft und dadurch kurzfristig zum Tod führen kann, oder wenn es sich um ein Kind handelt, das den tödlichen Preis voraussichtlich erst in einigen Jahrzehnten zu zahlen hat.

Solche wertenden Entscheidungen sind nicht nur auf normativer Ebene zu treffen, sondern auch bezüglich der Einschätzung der lebensweltlichen Situation. Unterschiedliche Einschätzungen können zu einer diametral entgegengesetzten ethischen Bewertung führen. Wenn man, worauf die einschlägige Forschung hindeutet, davon ausgeht, dass der Beitrag von Schulschließungen zur Eindämmung der Pandemie eher gering ist, steht einem kleinen Nutzen ein großer Schaden gegenüber. Unter dieser Prämisse sind Schulschließungen aus utilitaristischer Sicht nicht zu rechtfertigen. Hält man diesen Effekt für größer, oder die durch die Schulschließungen verursachten Schäden für reversibel oder weniger schwerwiegend, waren hingegen aus utilitaristischer Sicht Schulschließungen geboten.

## Fazit

Die Frage, ob Schulschließungen zur Bekämpfung der Pandemie ethisch zulässig waren, kennt keine einfache Antwort. Das liegt nicht nur an der Komplexität und zumindest anfänglichen Unübersichtlichkeit der Situation. Es liegt auch daran, dass Menschen unterschiedliche Werte und ethische Theorien bevorzugen sowie zu unterschiedlichen Bewertungen der Realität kommen.

Aus deontologischer Sicht ist eine ethische Rechtfertigung der Schulschließungen kaum zu gewinnen. Dazu müsste man nicht nur begründen, warum ausnahmsweise doch eine Verletzung der einen zum Wohle der anderen zulässig sein sollte. Man müsste auch zeigen, dass Schulschließungen einen nennenswerten Beitrag zum Gesundheitsschutz für Dritte leisten, also überhaupt die Erfüllung einer moralischen Pflicht darstellen. Das ist jedoch sehr ungewiss. Somit ist es wenig plausibel, hier eine Pflichtenkollision anzunehmen. Tendenziell kommen deontologische Betrachtungen daher zu dem Ergebnis, dass die Schulschließungen unethisch waren.

Ausgangspunkt einer utilitaristischen Bewertung ist eine Kosten-Nutzen-Analyse der Schulschließungen. Angesichts einer Vielzahl von Faktoren, die höchst unterschiedlich bewertet werden, können Schulschließungen aus dieser Perspektive grundsätzlich sowohl befürwortet als auch abgelehnt werden. Allerdings spielt der ungewisse Zusammenhang zwischen Schulschließungen und Eindämmungseffekt auch für eine utilitaristische Bewertung eine wichtige Rolle. Um einen sicheren Schaden für die einen mit einem ungewissen Nutzen für die anderen zu rechtfertigen, müsste der potenzielle Nutzen den definitiven Schaden sehr deutlich überwiegen.

Auch wenn man angesichts der Herausforderung und existentiellen Bedrohung durch die Pandemie Schulschließungen insbesondere im ersten Halbjahr 2020 als vertretbares Mittel der Pandemiebekämpfung betrachtet, so wird doch deutlich, dass diese Entscheidung ethisch zumindest prekär ist. Daher stellt sich, nicht zuletzt mit Blick auf zukünftige Herausforderungen, die Frage, wie die „ethische Bilanz“ dieser Situation zu verbessern ist. Ungeachtet unterschiedlicher ethischer Theorien und Bewertungen der Realität kann hier ein mit dem Präferenzutilitarismus und epistemologisch gewonnener Impuls aufgenommen werden, der für einen größeren individuellen Entscheidungsspielraum plädiert (Schulze Heuling 2020).

Konkret würde das bedeuten, dass Kinder und Jugendliche bzw. ihre Eltern selbst entscheiden, ob sie den Präsenzunterricht besuchen möchten oder nicht. Das mag aus bildungswissenschaftlicher oder pädagogischer Perspektive zunächst befremdlich erscheinen. So besteht die berechtigte Sorge, dass möglicherweise gerade jene fernbleiben, die besonders vom Unterrichtsbesuch profitieren würden. Daneben ist unklar, wie möglicherweise entstehende bzw. sich verstärkende Disparitäten aufgefangen werden sollen. Zugleich sollten realistische Handlungsoptionen, zumal in krisenhaften Situationen, nicht mit Idealsituation kontrastiert werden (Demsetz 1969). Insofern ist es unter gegebenen Umständen vorzugswürdig, den Unterrichtsbesuch zu ermöglichen, als diesen entweder für alle zu verunmöglichen oder aber alle zur Teilnahme zu zwingen und damit einige einem großen Gesundheitsrisiko auszusetzen oder starke Ängste auszulösen.

Auch deontologisch betrachtet, ist diese Lösung bestechend, da mangels erzwungener Schulschließungen oder erzwungenen Schulbesuchs keine Verletzungshandlungen vorliegen. Ein freiwilliges Fernbleiben vom bzw. Teilnehmen am Präsenzunterricht ist ethisch unproblematisch. Maßnahmen, die Dritte schützen wie etwa Abstand gegenüber Lehrkräften oder das Tragen von Masken beim Besuch der Großeltern, werden dadurch nicht verunmöglicht.

Für eine utilitaristische Betrachtung wäre die Wahlmöglichkeit insofern vorteilhaft, als dass der Schaden, den eine individuell abgewogene Entscheidung verursacht, geringer als bei einer allgemeinen Schulschließung ausfallen dürfte. Wenn zugleich die Auswirkungen eines freiwilligen Schulbesuchs auf das Infektionsgeschehen genau beobachtet werden, wäre bei Bedarf ein schnelles Eingreifen weiterhin möglich. Zugleich wäre damit womöglich ein Erkenntnisgewinn verbunden, der nicht nur das Niveau ethischer Diskussionen über Schulschließungen heben, sondern die Politik der Pandemiebekämpfung insgesamt verbessern könnte. Dieses Ziel ist aus jedweder ethischen Perspektive unterstützenswert.

## Supplementary Information





## Footnotes

1 Bei den Studien handelt es sich ausschließlich um Schülerleistungsstudien, die mit standardisierten Assessments – meist in den Domänen Mathematik, Lesen bzw. Muttersprache – die fachlichen Schülerleistungen (meist in Anlehnung an den Kompetenzbegriff nach Weinert oder den Literacy-Begriff der OECD) erfassen. Während rund die Hälfte der Studien (siehe Helm et al. 2021) über ein echtes Längsschnittdesign verfügt, das erlaubt, individuelle Lernverläufe während des Fernunterrichts zu modellieren und mit früheren Lernverläufen zu vergleichen, versuchen andere Studien mit einem querschnittlichen Kohortendesign (Vergleich der Schülerleistungen der Corona-Kohorte mit früheren Kohorten) das Auslangen zu finden. Neben dem Fokus auf „Hauptfächer“ ist einschränkend auch darauf hinzuweisen, dass in den meisten Studien keine repräsentative Stichprobe vorliegt; insbesondere bildungsbenachteiligten Schüler*innen dürften unterrepräsentiert sein. Für nähere Details zu den Studiendesigns siehe bspw. König und Frey (2022).

2 Vgl. Helm et al. (2021) für eine umfassende Liste von Studien.

3 Vgl. Helm et al. (2021) für eine umfassende Liste von Studien.

4 z. B.: https://www.derstandard.at/story/2000121579081/corona-experte-kinder-spielen-eine-nicht-so-bedeutende-rolle-in (aufgerufen am 28.05.2022), https://www.deutschlandfunkkultur.de/kinderarzt-jakob-maske-kitas-und-schulen-muessen-offen-100.html (aufgerufen am 27.07.2022).

5 Schulschließungen sind auch eine Herausforderung für die pädagogische Ethik. Die ethische Bewertung von Schulschließungen sollte daher auch vor dem Hintergrund der Pädagogischen Ethik erfolgen. Wir leisten dies an dieser Stelle nicht, weil wir im vorliegenden Beitrag nicht das didaktische Handeln von Lehrkräften (das in der Pädagogischen Ethik im Fokus steht) in den Blick nehmen, sondern eine vordergründig gesundheitspolitische Maßnahme, die zur Eindämmung der Pandemie gedacht war. Allerdings geben wir einen ersten Anstoß zur pädagogisch ethischen Bewertung von Schulschließungen im Online-Zusatzmaterial.
