# Supplementary material for: School Closures - an Ethical Challenge
Source: Z Bild Forsch. 2022 Oct 10;12(3):631–45. [Article in German] doi: 10.1007/s35834-022-00364-4 (PMC9549434; doi:10.1007/s35834-022-00364-4)
Supplement: Supplementary file 1 [file 35834_2022_364_MOESM1_ESM.docx]

Zeitschrift für Bildungsforschung

**Schulschließungen als ethische Herausforderung**

Zusatzmaterial

Dagmar Schulze Heuling

Christoph Helm

***Ergänzung 1***

Review-Studien (z.B. Walsh et al., 2021; Tan, 2021) zeigen, dass die Befundlage zu dieser Frage heterogen und inkonsistent ist. Auf Ebene von Einzelstudien beobachten Staguhn et al. (2021) positive Effekte von Schulschließungen auf COVID-19-Infektionsraten. Dagegen liegen zwei Studien (von Bismarck-Osten et al., 2021; Isphording et al., 2021) aus Deutschland vor, die keinen Anstieg der Infektionsraten bei Schulwiedereröffnung beobachten können. Letztere werden von internationalen Review-Studien (Ludvigsson, 2020; Irfan et al., 2021) gestützt, die zu dem Schluss kommen, dass es unwahrscheinlich ist, dass Kinder die Treiber der Pandemie sind. Die Studienautor*innen argumentieren daher, dass (a) die Schließung/Wiedereröffnung von Schulen und Kindergärten keinen relevanten Einfluss auf die COVID-19-Sterblichkeitsrate bei älteren Menschen haben dürfte und (b) für Kinder (<10 Jahre) es weitgehend sicher ist, die Schule zu besuchen. Überhaupt geht von COVID-19 keine nennenswerte Gesundheitsgefahr für Kinder aus, wie Meta-Studien belegen (z.B. Hoang et al., 2020; Liu et al., 2020). Dagegen sind Schulschließungen nicht ohne gesundheitliche Folgen (Tan, 2021; Viner et al., 2020).

***Ergänzung 2***

Der 7-Ländervergleich „Europas Schulen in der Corona-Pandemie“ (Freundl et al., 2021) des ifo München zeigt, dass Frankreich gemeinsam mit Spanien und Schweden die Schulen an vergleichsweise wenigen Tagen (teilweise) geschlossen hatte (Frankreich: 40 Tage vollständig/16 Tage teilweise; Deutschland 74/109).

Was Schülerleistungsstudien zu Leistungseinbußen während der Schulschließungen betrifft, haben wir einen relativ guten Überblick durch eine Vielzahl von einschlägigen Meta-Reviews und Meta-Analysen (z.B. Zierer, 2021; Hammerstein et al., 2021; König & Frey, 2022; Patrinos & Donelly, 2021; …). Keine dieser Meta-Studien enthält Studien zu Frankreich, da sie nur deutsch und/oder englischsprachige Publikationen berücksichtigen.

Allerdings liegt die in französischer Sprache veröffentliche Studie von Andreu et al. (2020) vor. Freundl et al. (2021) fassen die Ergebnisse wie folgt zusammen: „Zudem wurden standardisierte Schülerleistungstests, die in den französischen Grundschulen im Januar 2020 sowie 2021 durchgeführt wurden, ausgewertet (Andreu et al. 2021). Die Ergebnisse deuten darauf hin, dass der Anteil der leistungsschwächeren Schüler*innen im Vergleich zum Vorjahr tendenziell zurückging; möglicherweise steht dies in Zusammenhang mit der vergleichsweise geringen Länge der Schulschließungen in Frankreich. Allerdings haben sich Schüler*innen aus weniger privilegierten Schulen im oben genannten Zeitraum weniger verbessert, was ihre Lesefähigkeit betrifft (Andreu et al. 2021).“ (Freundl et al., 2021, S. 46)

Der Befunde, dass Leseleistungen im Lockdown zunahmen, wurde auch in anderen Leseleistungsstudien beobachtet (siehe Helm et al., 2021). Eine mögliche Erklärung liegt darin, dass mehr Zeit für Lesen im Lockdown verwendet wurde und Lesen eine Domäne darstellt, die offenbar vergleichsweise leichter ohne direkte Anleitung durch die Lehrkraft erlernt werden kann.

Gleichzeitig zeigt sich für Frankreich – wie für viele andere Länder auch (vgl. Helm & Postlbauer, 2021; Wößmann et al., 2020, 2021) –, dass die schulischen Aktivitäten während der Schulschließungen stark zurückgingen: „Auch in Frankreich ergab eine Umfrage während des ersten Lockdowns (März bis Mai 2020) von Di Pietro et al. (2020) eine Reduktion der schulischen Aktivitäten von Grundschüler*innen um knapp die Hälfte der Zeit, verglichen mit einem regulären Schultag vor der Pandemie.“ (Freundl et al., 2021, S. 46)

Freundl et al. (2021) berichten internationale Vergleichszahlen, die Frankreich einen „hohen“ Digitalisierungsstand der Schulen während der Pandemie attestieren, sodass – zumindest im Vergleich zu Deutschland – Frankreich über eher förderlichere Rahmenbedingung für die Implementation von digitalem Unterricht zu verfügen scheint.

Was die Kompensationsmaßnahmen betrifft, haben unterschiedliche Länder sehr unterschiedliche Maßnahmen gesetzt, sodass ein Vergleich schwierig ist. Zu Frankreich’s Bemühungen berichten Freundl et al. (2021, S. 46):

„Frankreichs Regierung bemühte sich vor allem darum, benachteiligte Schüler*innen zu unterstützen. So stellte das Land beispielsweise zusätzliche Unterstützung für Haushalte mit niedrigerem Einkommen zu Verfügung, bemühte sich, Internetzugangsbarrieren zu beseitigen, und subventionierte die für Online-Unterricht notwendigen technischen Geräte. Des Weiteren wurde die Initiative »Devoirs faits« (dt. »erledigte Hausaufgaben«) ausgeweitet, welche Schüler*innen bei ihren Hausaufgaben unterstützt (OECD 2021b). Auch wurde, ähnlich wie in Deutschland, ein Ferienprogramm zur Unterstützung von Schüler*innen ins Leben gerufen. Zudem wurden etwa 5 000 zusätzliche Lehrkräfte eingestellt, um Abwesenheiten von an Covid-19 erkrankten Lehrkräften auszugleichen (OECD 2021a).“

Das Fazit, das aus diesem Ländervergleich für unseren Beitrag gezogen werden kann, ist, dass auch Länder mit nur einer Schulschließung zumindest für den ersten Lockdown mit ähnlichen Herausforderungen (z.B. Rückgang der Lernzeit, Anstieg der Bildungsungleichheit, Mehrfachbelastung der Eltern) zu kämpfen hatten als Länder mit mehreren Lockdowns.

Freundl et al. (2021) differenzieren allerdings nicht zwischen Ländern mit einer oder mehreren Schulschließung(en), wenn es um die kritische Beurteilung eben dieser Schließungen geht: „Hierbei sollte erwähnt werden, dass Schulöffnungen diversen Erkenntnissen zufolge keinen Treiber der Pandemie darstellten (Ludvigsson 2020; Viner et al. 2020; Isphording et al. 2021; von Bismarck-Osten et al. 2021). Umso bedenklicher ist es daher, dass die Belange der Schüler*innen während der Pandemie hintenangestellt wurden.“ (Freundl et al., 2021, S. 48)

Vor diesem Hintergrund und vor dem Hintergrund des knappen Platzes verzichten wir auf eine umfassend Aufnahme dieses Ländervergleichs. Ein solcher würde unsere Argumentation/Darstellung weder unterstützen noch untergraben.

***Über das Manuskript hinausgehende Literatur***

Andreu, S., Cioldi, I., Conceicao, P., Eteve, Y., Fabre, M., Le Breton, S., Persem, E., Portelli, T., Rue, G., Rocher, T., Vourc’h, R. und Wuillamier, P. (2021), *Evaluations 2021 Point d’étape CP: premiers résultats,* Ministère de l’Éducation nationale, de la jeunesse et des sports, Paris.

Freundl, V., Stiegler, C., & Zierow, L. (2021). Europas Schulen in der Corona-Pandemie – ein Ländervergleich. *Ifo Schnelldienst, 74*(12), 41–50.

Helm, C. & Postlbauer, A. (2021). Schulschließungen in Österreich – Ein Fazit nach einem Jahr Pandemie. *Psychologie in Erziehung und Unterricht, 68*(4), 306–311. https://doi.org/10.2378/peu2021.art27d

Wößmann, L., Freundl, V., Grewenig, E., Lergetporer, P., Werner, K., & Zierow, L. (2020). Bildung in der Coronakrise: Wie haben die Schulkinder die Zeit der Schulschließungen verbracht, und welche Bildungsmaßnahmen befürworten die Deutschen? (No. 9). *ifo SCHNELLDIENST.*

Wößmann, L., Freundl, V., Grewenig, E., Lergetporer, P., Werner, K., & Zierow, L. (2021). Bildung erneut im Lockdown: Wie verbrachten Schulkinder die Schulschließungen Anfang 2021? (No. 5). *ifo SCHNELLDIENST.*

***Ergänzung 3***

Neben der philosophischen Ethik bietet sich für die Beurteilung/Bewertung von Schulschließungen auch die Pädagogische Ethik an. Die Pädagogische Ethik stellt laut Drerup (2019) und Reitemeyer (2020) eine wenig entwickelte Teildisziplin der Ethik dar. Ihr Anwendungs- bzw. Aufgabenbereich hängt laut Drerup (2019) stark davon ab, wie „weit oder eng man den Begriff des ‚Pädagogischen‘“ bestimmt. So kann unter einem sehr weiten Verständnis von Pädagogischer Ethik „die systematische Reflexion von ethischen Problemen und Begründungsfragen, die sich im Rahmen pädagogischer Konstellationen (z. B. Sozialpädagoge-Klient), Institutionen (z. B. Schule; Heim) und (Semi-)Professionen (z. B. Lehrerberuf) ergeben“ (Drerup, 2019, S. 226), verstanden werden. In einem eher engen Verständnis, kann unter „Pädagogischer Ethik hingegen ein ‚eigener Bereich der Reflexion‘ [Oelkers, 1992, S. 12], verstanden [werden], der sich mit der Ethik des Lehrens beschäftigt und mit der Frage, ob es so etwas wie spezifische moralische Grundsätze für pädagogisch Handelnde gibt, denen Bildungsziele, Lehrinhalte, Unterrichtsmethoden, administrative Entscheidungen sowie der Lehrende als Lehrender selbst unterworfen sind“ (Reitemeyer, 2020, S. 4). Ein solcher moralischer Grundsatz ist laut Reitemeyer (2020) bspw., dass bei allen didaktischen Entscheidungen auf die Würde, den freien Willen und die Persönlichkeit der Lernenden im besonderen Maße geachtet wird, bspw. in dem Unterrichtsmethoden ausgeschlossen werden, die physische oder psychische Gewalt ausüben (z.B. an den Pranger stellen, unverstandenes Wissen indoktrinieren), und Methoden präferiert werden, die „allen Lernenden eine gleichberechtigte Teilnahme am Unterricht eröffnen und dies unabhängig vom Stand ihres Vorwissens, ihrer Herkunft, ihrer Religion, ihres Geschlechts oder ihrer Hautfarbe usw.“ (S. 9).

Für die pädagogisch ethische Prüfung/Reflexion von Schulschließungen bietet sich vor allem diese engere Definition von Pädagogischer Ethik an, da sie mit dem Fokus auf den ethischen Gehalt didaktischer Entscheidungen jene didaktischen Aufgaben in den Blick nimmt, die während der Schulschließungen von den Lehrkräften anfangs nicht und später nur digital im Distance Learning umgesetzt werden konnten bzw. von den Eltern übernommen werden mussten (z.B. Helm et al., 2021; Huber et al., 2020). Man könnte die pädagogisch ethische Prüfung von Schulschließungen daher an der Frage festmachen, inwiefern Schulschließungen dazu beigetragen haben, dass der ethische Gehalt von didaktischen Entscheidungen und Maßnahmen im Distance Learning zu- oder abgenommen hat. Oder anders formuliert: Wurde die Einhaltung moralischer Grundsätze durch pädagogisch Handelnde – das waren während der Schulschließungen nicht nur die Lehrkräfte, sondern häufig auch die Eltern, (ältere) Geschwister und Angehörige – durch die flächendeckenden Schulschließungen gestärkt oder untergraben?

Eine solche Reflexion der von Reitemeyer (2020, S. 5) angeführten fünf „Aufgaben pädagogischer Ethik“ (= pädagogisch ethische Prüfung von Bildungsziele, Lehrinhalte, Unterrichtsmethoden, administrative Entscheidungen, Lehrender selbst) bedarf einer eigenen theoretisch und empirisch gestützten wissenschaftlichen Diskussion. An dieser Stelle kann eine entsprechende Reflexion nur an einem Beispiel angedeutet werden:

Reitemeyer (20202, S. 6) argumentiert im Rahmen der pädagogisch ethischen Prüfung von Bildungszielen, dass Lehrende nicht nur die in Curricula verankerte Ausbildungs- oder Studierfähigkeit der Lernenden vor Augen haben sollten, „sondern auch die Förderung ihrer individuellen Begabungen und Interessen unter Berücksichtigung ihrer charakterlichen Besonderheiten. Denn dann stehen die Chancen gut, dass nicht nur die Schulbildung erfolgreich abgeschlossen wird, sondern darüber hinaus ein selbständig urteilender junger Erwachsener die Schule verlässt, der seine erworbenen Kompetenzen für sich selbst und seinen weiteren Lebensweg gut nutzen kann, aber auch in den Dienst der Gesellschaft und des Rechtsstaats stellt.“ (S. 6) Hier wird die Individualisierung als pädagogische (und ethische) Aufgabe angesprochen.

Nun können Argumente für und gegen eine verstärkte/verringerte Umsetzung von Individualisierung im Lehr-Lernprozess während des Distance Learnings (und damit für die Einhaltung dieses spezifischen pädagogisch ethischen Grundsatzes) ins Feld geführt werden. Tatsächlich wird in der empirischen Literatur die verstärkte Individualisierung als eine – aus Sicht von Schüler*innen wahrgenommene – „Errungenschaft“ von Schulschließungen beschrieben (z.B. Schneider et al., 2022, S. 239f.):

Vor allem die Schülerinnen und Schüler selbst geben an, den Zuwachs an Autonomie und Selbstständigkeit beim Lernen zu schätzen und »… eher selbst nach Lösungswegen [zu suchen], statt den Lehrer direkt zu fragen« (Schul- Barometer, SuS, ID 1526, v_ 474, D). Dabei schätzen viele von ihnen am Fernunterricht besonders, dass sie ihre Zeit nach ihrem individuellen Lernrhythmus selbstständig einteilen und dadurch produktive Zeiten am Tag effizienter nutzen könnten: »Man kann sich seinen Tag einteilen wie man möchte; wenn man in der Früh/ am Vormittag produktiver ist, kann man seine Aufgaben gleich am Morgen erledigen, wenn man aber der Typ ist, der erst am Nachmittag oder Abend seine Höchstleistungen erbringt, dann kann man in dieser Zeit seine Aufgaben erfüllen« (Schul- Barometer, SuS, ID 4445, v_ 464, Ö). »Produktive Phasen [können dadurch] effizienter genutzt werden« (Schul- Barometer, SuS, ID 3946, v_ 464, Ö). Allerdings könne diese Möglichkeit der freien Zeiteinteilung laut der Aussage einer Schülerin bzw. eines Schülers »mit fehlender Motivation wieder zur negativen Konsequenz« (Schul- Barometer, SuS, ID 3731, v_ 464, Ö) werden, insbesondere dann, wenn Schülerinnen und Schüler Schwierigkeiten bei der Motivation und selbstständigen Organisation des Tagesablaufs haben. Viele Schülerinnen und Schüler betonen zudem, dass sich der Fernunterricht positiv auf ihren Schlafrhythmus auswirke, sie mehr Schlaf bekämen und dadurch mehr Energie für die Schulaufgaben hätten: »Man kann ausschlafen, was zu besserer Konzentration führt …« (Schul- Barometer, SuS, ID 5027, v_ 464, D).

Auch anhand der offenen Angaben der Eltern bestätigt sich der Befund, dass der Fernunterricht neue, bzw. erweiterte Möglichkeiten für die Förderung des selbstständigen Lernens aufgezeigt hat. Kinder würden laut Angaben einiger Eltern lernen, sich besser zu disziplinieren und zu planen, wovon sie auch im späteren Leben sehr profitieren könnten: »meine Tochter lernt sich den Stoff bzw. die Arbeitsaufgaben selbst einzuteilen, was ihr später z.B. [in der] Universität zugutekommen wird.« (Schul- Barometer, Eltern, ID 3143, v_ 570, Ö). Dabei würden die Selbstständigkeit und Selbstdisziplin der Kinder und Jugendlichen wesentlich gefördert: »Die Einteilung der Hausaufgaben fällt von Tag zu Tag immer leichter. Und wenn sie zum Schluss den Button ›Abgeben‹ drücken, dann erfüllt sie dieser Moment mit Stolz« (Schul- Barometer, Eltern, ID 4372, v_ 570, Ö).

Als Gegenargument kann eingeführt werden, dass dieses Potential des Distance Learning hinsichtlich Selbständigkeit und Individualisierung nicht von allen Schüler*innen gleichermaßen genutzt wird/werden kann. Wie im Zitat von den Schüler*innen selbst angesprochen, braucht es im Distance Learning deutlich mehr Motivation (siehe dazu auch Huber et al., 2020). Darüber hinaus muss auch das selbstständige Lernen und insbesondere die Individualisierung didaktisch sinnvoll angeleitet werden, was wiederum den Eltern didaktische und fachliche Fähigkeiten abverlangt, die gerade bei Eltern aus sozioökonomisch schwächeren Schichten und Eltern mit geringerem Bildungsabschluss häufig nicht gegeben waren/sind (z.B. Helm et al., 2021; Porsch & Porsch, 2020). Es ließen sich viele weitere Rahmenbedingungen (z.B. die digitale Ausstattung von Lehrenden und Lernenden) anführen, von denen eine qualitätsvolle Umsetzung der Individualisierung (aber auch andere pädagogisch ethische Grundsätze) abhängt, sodass eine pädagogisch ethische Prüfung der didaktischen Aufgaben im Distance Learning kein leichtes Unterfangen darstellt.

Es ist aber grundsätzlich höchst fraglich, ob didaktische Aufgaben von Eltern – also Laien, oft ohne jegliche didaktische Vorbildung – in qualitätsvoller Weise ausgeführt werden können, sodass eine umfassende, theoretisch und empirisch gestützte Reflexion der pädagogisch ethischen Qualität der Bewältigung didaktischer Aufgaben sehr wahrscheinlich zum Schluss kommen würde, dass Schulschließungen gerade auch vor diesem Hintergrund nicht/kaum argumentierbar sind.

Darüber hinaus kann auf Systemebene eingewendet werden, dass Schulschließungen dem pädagogisch ethischen Grundsatz „allen Lernenden unabhängig ihrer (sozialen) Herkunft eine gleichberechtigte Teilnahme am Unterricht zu eröffnen“ zuwiderläuft, da empirisch wiederholt nachgewiesen wurde, dass gerade Schüler*innen aus sozial schwachen Schichten im Distance Learning – aus ein Vielzahl von Gründen – auf der Strecke blieben (z.B. Helm et al., 2021).

***Über das Manuskript hinausgehende Literatur***

Drerup, J. (2019). Pädagogische Ethik. In J. Drerup & G. Schweiger (Hrsg.), *Handbuch Philosophie der Kindheit* (S. 225–235). J.B. Metzler. https://doi.org/10.1007/978-3-476-04745-8_29

Helm, C., Huber, S. G. & Loisinger, T. (2021). Was wissen wir über schulische Lehr-Lern-Prozesse im Distanzunterricht während der Corona-Pandemie? – Evidenz aus Deutschland, Österreich und der Schweiz. *Zeitschrift für Erziehungswissenschaft,* 1–75. https://doi.org/10.1007/s11618-021-01000-z

Huber, S. G., Günther, P. S., Schneider, N., Helm, C., Schwander, M., Schneider, J., & Pruitt, J. (2020). *COVID-19 und aktuelle Herausforderungen in Schule und Bildung.* Waxmann. https://doi.org/10.31244/9783830942160

Porsch, R., & Porsch, T. (2020). Fernunterricht als Ausnahmesituation: Befunde einer bundesweiten Befragung von Eltern mit Kindern in der Grundschule. In D. Fickermann & B. Edelstein (Hrsg.), *Die Deutsche Schule. „Langsam vermisse ich die Schule …“: Schule während und nach der Corona-Pandemie* (S. 61–78). Waxmann.

Reitemeyer, U. (2020). *Pädagogische Ethik.* https://www.uni- muenster.de/imperia/md/content/ew/forschung/feuerbach/padagogische_ethik.pdf [19.08.2022]

Schneider, J., Huber, S. G., & Berger, J. (2021). COVID- 19 und Bildung: Chancen und Errungenschaften in der Krise. In S. G. Huber (Hrsg.), *Jahrbuch Schulleitung: Vol. 2021. Impulse aus Wissenschaft und Praxis* (S. 245–259). Carl Link.
